# Estimating the size of the homeless adolescent population across seven cities in Cambodia

**DOI:** 10.1186/s12874-017-0293-9

**Published:** 2017-01-26

**Authors:** Lindsay Stark, Beth L. Rubenstein, Kimchoeun Pak, Rosemary Taing, Gary Yu, Sok Kosal, Leslie Roberts

**Affiliations:** 1 Department of Population and Family Health, Mailman School of Public Health, Columbia University, 60 Haven Avenue, New York, NY 10032 USA; 2 Department of Epidemiology, Mailman School of Public Health, Columbia University, New York, USA; 3 Moulathan Consulting, Phnom Penh, Cambodia; 4 Friends International, Phnom Penh, Cambodia; 5 New York University Rory Meyers College of Nursing, New York, USA; 6 National Institute of Statistics, Ministry of Planning, Royal Government of Cambodia, Phnom Penh, Cambodia

**Keywords:** Homeless, Adolescents, Capture-recapture, Cambodia

## Abstract

**Background:**

The Government of Cambodia has committed to supporting family care for vulnerable children, including homeless populations. Collecting baseline data on the numbers and characteristics of homeless adolescents was prioritized to illuminate the scope of the issue, mobilize resources and direct the response.

**Methods:**

Administrative zones across seven cities were purposively selected to cover the main urban areas known to have homeless populations in Cambodia. A complete enumeration of homeless individuals between the ages of 13 and 17 was attempted in the selected areas. In addition, a second independent count was conducted to enable a statistical estimation of completeness based on overlap across counts. This technique is known as capture-recapture. Adolescents were also interviewed about their schooling, health and other circumstances.

**Results:**

After adjustment by the capture-recapture corrective multipliers (range: 3.53 -27.08), the study yielded an estimate of 2,697 13–17 year old homeless adolescents across all seven cities. The total number of homeless boys counted was significantly greater than homeless girls, especially in older ages.

**Conclusions:**

To the authors’ knowledge, this is the first time capture-recapture methods have been applied to a homeless estimation of this scale in a resource-limited setting. Findings suggest the number of homeless adolescents in Cambodia is much greater than one would expect if relying on single count data alone and that this population faces many hardships.

## Background

“Leave no one behind” is a central guiding principle of the post-2015 global development agenda. The international community is increasingly embracing the importance of inclusiveness and equity in policies and programming. However, actors are recognizing that the global monitoring framework lacks mechanisms to assess the most vulnerable and hard to reach populations [1]. For example, it is estimated that household surveys such as the Demographic and Health Survey (DHS) and the Multiple Indicator Cluster Survey (MICS) may overlook up to a quarter of the poorest wealth quintile [2]. Many of these missing millions are adolescents who are homeless or migrants living in improper housing, such as in construction sites or in urban slums.

Young people who lack a safe, stable and protective home face a multitude of adversities associated with extreme poverty. These adverse childhood experiences are linked to toxic levels of stress and impede proper physical, intellectual and emotional growth across the life course [3–5]. Scientific evidence about the specific impact of homelessness in childhood is limited, but some studies suggest that homeless adolescents have heightened risks, such as substance abuse, gang membership and commercial sex work, compared to adolescents living in homes [6–8]. Further, the disadvantages of childhood adversity persist with time. As children who experience adverse events in early life become adults, they are more likely to have poor health and limited productivity [3, 9].

Recently the Royal Government of Cambodia committed to investing in family strengthening initiatives and reducing the number of children outside of family care, including homeless populations. Before rolling out programs associated with this initiative, collecting baseline data on the numbers and characteristics of homeless children was needed to illuminate the scope of the issue, mobilize resources and direct the response.

This article describes the methodology and findings from an estimation of homeless adolescents in seven major cities in Cambodia led by the Cambodian National Institute of Statistics, Columbia University and Friends International, with support from Moulathan Consulting. Using a measurement technique known as capture-recapture, two independent counts were conducted on two different days in order to enable statistical estimates of completeness. A similar method was used in Brazil and Malawi to estimate the number of street children in select cities, but the definition of the target population was loosely defined and included children who worked on the street, yet slept in proper housing. Further, the Brazil and Malawi studies were limited to just one or two cities [10–12]. To the authors’ knowledge, this is the first time capture-recapture methods have been applied to a homeless estimation of this scale in a resource-limited setting.

## Methods

### Sampling

**Fig. 1:**
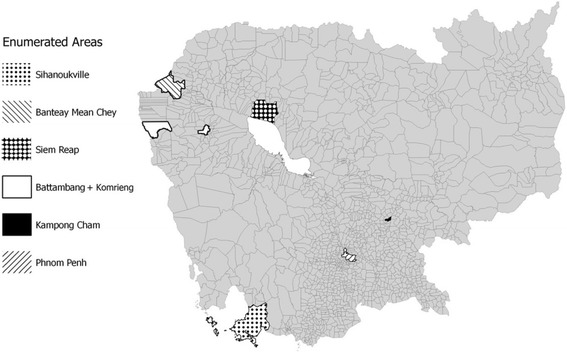
Map of enumerated districts

Fifteen administrative zones across seven cities were purposively selected to cover the main urban areas known to have young homeless populations, as determined by key informant interviews with local NGOs in the Cambodian Street Child Network and representatives from the Ministry of Social Affairs, Veterans and Youth Rehabilitation (MoSVY) (see Figure 1). NGO program data were also reviewed. In Phnom Penh, selection was done at the sangkat level (equivalent to boroughs) and elsewhere, selection was done at the district level (equivalent to counties). Within the selected zones, data collectors created maps to identify key sub-areas to visit and count adolescents. Within those sub-areas, data collectors attempted to enumerate all homeless 13–17 year olds.

### Inclusion criteria

Enumeration of homeless children is complicated by the definition of homeless [13]. For example, individuals who sleep in a shelter made of plastic tarps may not self-identify as homeless. Adolescents who work on the street to earn income on a daily basis are often mistakenly classified as street-living by observers. In this study, adolescents were classified as homeless if they were under 18 years of age and, during the week preceding data collection, they always or sometimes lived on the street, in emergency shelters or in public places, including construction sites (according to self-report). Adolescents living in dwellings with daily rent were included because the risk of eviction in these situations was extremely high. Adolescents were also classified as homeless if they slept in dwellings that did not offer approximately 3.5 square meters of covered space per person and did not provide basic protection from the elements [14]. Insufficient protection from the elements was operationalized to mean two or more walls made out of makeshift plastic sheeting. Boat-living children were included based on the size of the living space and the adequacy of the construction, as previously defined [14].

The inclusion criteria were established based on international standards, field observations, and conversations with NGO outreach workers, government officials and the inter-agency technical working group guiding the project. Homeless status was based on the adequacy of adolescents’ *current* living situation. Those adolescents who may have proper homes in rural areas, but who had migrated to cities, were therefore evaluated based on their current living situation in the city.

### Study design

Two separate teams of enumerators conducted two independent counts of homeless 13–17 year olds in the key sub-areas of the selected administrative zones. The counts were conducted on two separate, full days, from morning through evening. Within these sub-areas, enumerators approached any young person thought to meet the age and inclusion criteria, introduced themselves and asked the young person to describe his/her housing situation. Those young people who were homeless and between the ages of 13 and 17 were then interviewed to gather identifying data, as well as information about their circumstances, including schooling, literacy, health, caregiver status and general well-being.

To avoid duplicate counting, all adolescents were shown a unique cartoon. If the adolescent told the enumerator that s/he had already been shown the same cartoon earlier in the day, the individual was not counted again. Different cartoons were used on each day and adolescents counted on the second day were asked to recall the previous day’s cartoon to facilitate the detection of overlap between the two counts. Identifying data (e.g., name, age, province of origin) were also used to detect overlap between the two counts. The study team used the overlap between the two independent counts to approximate the extent to which the first count may have missed individuals and create a corrective multiplier. This technique is known as capture-recapture (Stephen, 1996; Lum et al., 2013).

Data collection took place over four-weeks between August and September 2015. All data were collected electronically using the Field Task application for smart phones [15].

### Ethical considerations

The team spent considerable time and effort designing protocols to protect the study participants. The minimum age of 13 years for respondents was selected based on extensive conversations with social workers in Cambodia who work with the target population and regularly observe children’s decision-making skills. The minimum age of 13 years is also consistent with international recommendations, which state that adolescents’ ability to meaningfully participate in the consent process is informed by their cultural and experiential context [16, 17].

To ensure confidentiality of the identifiable data, data were only accessible to research team leads on a password-protected server. All identifying information was deleted after matching was complete. Still, despite these precautions, there remained a small risk that confidentiality would be breached, hence the requirement of informed consent and the restriction against participation by children under 13 years of age.

All data collectors were hired through partnerships with local NGOs in the Cambodian Street Child Network and thus previously had substantial experience working with the target population. Data collectors gave a small snack to all children who were encountered, whether or not they consented to participate in the research. Ethical approval was obtained from the Institutional Review Board at Columbia University (AAAP2507) and the Cambodian National Ethics Committee.

### Statistical analysis

Chi-squared tests and t-tests were used to compare the characteristics of adolescents across sex and age. For capture-recapture, the final estimate of adolescents not included in either list was calculated by multiplying the first count of adolescents by the second count of adolescents, and then dividing this product by the number of matched respondents. The total estimate is the sum of the adolescents in the first count, the adolescents in the second count, the adolescents in both counts and the adolescents not included in either list. All descriptive statistics were calculated using R [18] and SAS 9.4 [19].

For the purpose of this study, matching was achieved by comparing the following variables: family name, given name, nickname, sex, age, parents’ names and province of origin. Duplicates and matches were identified through three techniques and searched across nearby districts or sangkats to allow for the possible movement of adolescents. First, the CRAN RecordLinkage package in R was used to match cases with weights greater than or equal to 0.80, as determined by the package algorithm. (The algorithm examines the degree of differentiation provided by each response in an observation, and if that value matches a response for the same variable in another observation, the package adds the degree of differentiation between the two observations to the overall measure of matching, called a “weight.”) Second, all remaining observations were manually reviewed to identify matches that the package did not detect. Most manually detected matches that were missed by the package were due to limitations faced by data collectors in transcribing Khmer names to the Roman alphabet during electronic data entry. Specifically, the CRAN RecordLinkage package struggled to find some phonetic similarities that were masked by different spellings. A Research Assistant who was bilingual in English and Khmer assisted with the manual matching processes. Third, observations from adolescents who were able to successfully recall the Day 1 cartoon on Day 2 were manually reviewed to identify additional matches.

## Results

After capture-recapture adjustment, the study yielded an estimate of 2,697 homeless adolescents ages 13–17 across the seven cities where data collection was conducted. Across all areas combined, there were significantly more boys than girls counted (64.32% versus 35.68%, *p*-value <0.0001). The mean age was 14.37 years overall. On average, boys were two and one-half months older than girls (14.45 years versus 14.24 years, *p*-value <0.0671) (see Table 2).

In eight out of nine areas where capture-recapture was performed, the capture-recapture estimate was five or more times larger than the number of young people enumerated during Count 1. In Siem Reap, where match rates were the lowest, the capture-recapture estimate was 27 times the number of young people enumerated during Count 1 (see Table 1). These findings suggest that the population of homeless adolescents is significantly larger than enumerators were able to document on either of the individual counts^1^.

**Table 1:** Numbers of children by province and district, 13–17 year olds (capture-recapture)

Province	District/Sangkat	Count 1	Count 2	Matches	Total estimate
Banteay Meanchey	Krong Poi Pet	55	48	5	636
Battambang	Battambang	19	16	2	189
	Komrieng	11	20	3	107
Kampong Cham	Kampong Cham	22	19	4	150
Phnom Penh	Chamkar Mon	19	9	5	67
	Chbar Ampov	52	39	10	304
	Doun Penh	31	20	4	210
Preah Sihanouk	Preah Sihanouk	49	25	4	384
Siem Reap	Siem Reap	24	25	1	650
All areas		282	221	38	2,697

Among the 569 homeless adolescents interviewed (response rate = 96.30%), several patterns emerged (see Table 2). Almost all respondents reported having at least one parent alive. Furthermore, parents were reported to be the primary caregivers for 83.66% of respondents (79.78% for males and 90.59% for females, *p*-value = 0.0009), and most other respondents outside of parental care reported being cared for by other family members. Very few adolescents reported having no adult caregiver (3.02%), and all of the adolescents in this situation were male. Not having an adult caregiver was also more common amongst older adolescents (15–17 years), compared to younger adolescents (13–14 years) (5.06% versus 1.53%, *p*-value = 0.0157).

**Table 2:** Child characteristics, 13–17 year olds (*n* = 569, all districts)

	All	Male	Female	*p*-value
Sex	100%	64.32%	35.68%	<0.0001
missing = 0 children				
Age				
13 years	35.11%	34.63%	35.96%	0.1706
14 years	22.87%	20.50%	27.09%	
15 years	21.81%	21.88%	21.67%	
16 years	10.11%	11.36%	7.88%	
17 years	10.11%	11.63%	7.39%	
Missing = 5 children				
mean age, years (SD)	14.37 (1.32)	14.45 (1.37)	14.24 (1.23)	0.0671
missing = 5 children				
parental status				
both parents alive	79.43%	77.01%	83.74%	0.3102
only mother alive	14.36%	15.79%	11.82%	
only father alive	3.72%	4.16%	2.96%	
both parents deceased	2.13%	2.77%	0.99%	
don’t know	0.35%	0.28%	0.49%	
missing = 5 children				
current caregiver				
parent	83.66%	79.78%	90.59	0.0048
family member (not parent)	9.59%	10.53%	7.92	
acquaintance	1.95%	2.49%	0.99	
employer	0.53%	0.83%	0.00	
other	1.24%	1.66%	0.50	
no adult caregiver	3.02%	4.71%	0.00	
missing = 6 children				
school attendance				
every day	49.73%	45.83%	56.65%	0.0563
a few days a week	9.59%	10.00%	8.87%	
once in a while	3.02%	3.89%	1.48%	
never attend	37.66%	40.28%	33.00%	
missing = 6 children				
literacy				
able to read whole sentence	32.19%	27.25%	40.84%	0.0076
able to read parts of sentence	33.71%	34.13%	32.98%	
cannot read at all	33.33%	37.43%	26.18%	
other	0.19%	0.30%	0.00%	
don’t know	0.57%	0.90%	0.00%	
missing = 44 children				
work				
works 5 or more days per week and 5 or more hours per day	29.75%	32.33%	25.26%	0.0896
missing = 48 children				
chores				
does chores 5 or more days per week and 5 or more hours per day	6.60%	5.49%	8.56%	0.1775
missing = 54 children				
frequency that work/chores interfere with school				
always	8.25%	10.73%	3.89%	0.0361
sometimes	16.90%	16.40%	17.78%	
never	68.41%	65.62%	73.33%	
don’t know	6.44%	7.26%	5.00%	
missing = 72 children				
frequency that work/chores interfere with sleep				
always	8.00%	9.28%	5.76%	0.0383
sometimes	23.43%	26.35%	18.32%	
never	67.05%	62.57%	74.87%	
don’t know	1.52%	1.80%	1.05%	
missing = 44 children				
injured in the past 30 days (unable to work/study/do chores)				
yes	13.14%	15.57%	8.90%	0.0910
missing = 44 children				
sick in the past 30 days (unable to work/study/do chores)				
yes	29.14%	29.34%	28.80%	0.9785
missing = 44 children				
child feels safe where s/he lives				
very safe	44.57%	43.71%	46.07%	0.9225
somewhat safe	44.19%	44.91%	42.93%	
not at all safe	10.48%	10.48%	10.47%	
don’t know	0.76%	0.90%	0.52%	
missing = 44 children				
child trusts adults with whom s/he has contact				
a lot	48.57%	47.60%	50.26%	0.7620
somewhat	41.14%	42.51%	38.74%	
not at all	8.38%	7.78%	9.42%	
don’t know	1.90%	2.10%	1.57%	

About half of the respondents attended school on a daily basis, with girls attending school more regularly than boys (*p*-value = 0.0563). School attendance decreased as adolescents got older. Literacy was poor regardless of school attendance. Only about one third of the respondents could fully read the simple sentences presented to them, and one third of the respondents could not read at all. Again, girls’ literacy was significantly better than boys’ literacy (*p*-value = 0.0076) (see Table 2).

In terms of work, 29.75% of respondents reported working five or more hours per day on five or more days per week. Older adolescents worked more compared to younger adolescents (42.01% versus 20.86%, *p*-value < 0.0001), but did not differ significantly for boys compared to girls (32.33% versus 25.26%, *p*-value = 0.0896). In contrast to work, only 6.60% of adolescents reported a heavy burden of chores (five or more hours per day on five or more days per week). The percent of adolescents doing this amount of chores did not differ by age or across boys and girls (*p*-values = 0.8645 and 0.1775, respectively). One in four respondents reported that work or chores sometimes or always interfered with their schooling and nearly one in three reported that work or chores sometimes or always interfered with their sleep. Interference with schooling and sleep was significantly more common for boys, compared to girls (*p*-values = 0.0361 and 0.0383, respectively).

In the 30 days prior to being interviewed, 13.14% of respondents reported being unable to work, study or do chores due to injury and 29.14% reported being unable to work, study or do chores due to illness. Rates of illness were similar between boys and girls, but rates of injury appear to be higher amongst boys compared to girls (15.57% versus 8.90%, *p*-value = 0.0910). When asked to assess their sense of safety and trust, 10.48% of respondents said that they did not feel at all safe where they lived and 8.38% said that they did not have any trust in the adults with whom they interact. Older adolescents were significantly more likely to feel unsafe, compared to younger adolescents (14.86% versus 7.26%, *p*-value = 0.0049).

## Discussion

### Strengths

Globally, this is the first known study to attempt to apply a systematic and potentially reproducible method to simultaneously estimate young homeless populations in all the major urban areas within a country. Application of capture-recapture methods to the estimation of street children is relatively new. The research in Cambodia builds on recent work to estimate the population of street children in Brazil and Malawi using similar methods [10–12]. These findings, combined with the findings from Brazil and Malawi, demonstrate that capture-recapture can be feasibly carried out to measure young homeless populations in a range of settings.

An additional strength of this study is the use of clear inclusion criteria to define homelessness. Although the criteria diverge somewhat from commonly understood notions of street-living or street-working children, by focusing on young people’s housing situations as opposed to their daily activities (e.g., panhandling), this study is able to better target those adolescents who are likely missed by household surveys [20–22]. Furthermore, the housing-related inclusion criteria also facilitated the recognition of new populations of vulnerable adolescents not previously known to NGOs working with street populations, such as those living on construction sites.

Finally, having NGO workers serve as enumerators was important to the success of the effort. Not only were the NGO workers familiar with the local geography, but they also were able to establish trust and rapport with adolescents and families, as reflected by the 96.30% response rate.

### Fidelity to capture-recapture assumptions

The capture-recapture approach is not without limitations. First, capture-recapture assumes the population being counted is closed and that all members of the population can be matched if they appear in both counts. While key informants indicated that short-term movement in and out of the areas enumerated was low, there were likely at least a few children who entered or left the study area during the two-day data collection period. This movement would falsely inflate the estimates. In addition, there may have been some children who were counted twice but not identified as matches due to limitations in data quality or the computerized matching algorithm, which would also inflate the final estimates. The small sample size and single digit matches also affected the precision of the multipliers.

Second, capture-recapture assumes the lists being compared are independent (i.e., the biases within Count 1 and Count 2 are not related). While the use of outreach workers as enumerators was advantageous for the reasons described above, their involvement may have simultaneously undermined this assumption. Although enumerators were clearly instructed to cover areas beyond the places where they provide outreach services, a tendency to gravitate to familiar locations likely persisted. Moreover, it is likely that the areas where the enumerators gravitated were similar across counts, despite the fact that the teams were given strict guidance not to communicate their specific plans to their colleagues on other teams. These tendencies would falsely inflate the number of matches and lead to an underestimate of homeless children.

### Limitations

Data were not collected from children below 13 years due to ethical considerations. The study therefore lacks insights into children under 13. Future research is needed to get an indicative sense of whether capture-recapture for children under 13 years of age would yield similar multipliers to the 13–17 year olds or if the ratio of counted to uncounted children varies by age group.

In addition, the study was limited by self-reported measurements of adolescent characteristics. Suspicion of authority may have influenced the validity of responses to certain questions, especially those concerning trust and safety. Finally, NGO partners reported that staffing limitations likely limited the thoroughness of the enumeration.

### Policy implications

The study findings provide important information for practitioners and policymakers in Cambodia. Results suggest that the number of homeless children in Cambodia ages 13–17 is much greater than one would count in a single street census. In eight out of nine areas enumerated, Count 1 undercounted homelessness by a factor of five or more, indicating large numbers of vulnerable adolescents who are hidden from outreach workers and who may require social services. Conversations with partners confirmed that certain categories of adolescents covered by the study, such as those living on construction sites, were previously unknown to organizations and not currently receiving social services, despite apparent need.

More homeless boys were encountered than girls, especially in older ages. Further research is needed to understand what happens to homeless girls as they age and disappear from the population that was enumerated. The pathways and vulnerabilities faced by these uncounted girls, including in regard to sex work, require further illumination.

Overall, the interviews reflected the many hardships faced by homeless adolescents in Cambodia. Homeless adolescents often work long hours, are frequently injured, and do not get enough sleep. They are unable to pursue their education or attain basic literacy, and contend with frequent health problems that interfere with their day-to-day activities. An alarming number of adolescents do not feel safe or have relationships with any adults who they can trust. Still, despite these bleak circumstances, the vast majority of those interviewed said that they have a caregiver who is related to them, suggesting that one way to maximize the effectiveness of social services may be through family strengthening programs.

## Conclusion

In developing robust approaches to monitor young populations currently invisible to the system, Cambodia has the opportunity to systematically recognize and reach many of the most vulnerable members of society. Investments in children have been shown to catalyze long-lasting gains in families, communities and nations, but without inclusive data collection, these investments will not be appropriately targeted or evaluated [23, 24]. This study demonstrates that, with innovative methods, it is feasible to collect data on a hard to reach population. The findings are relevant to researchers, policymakers, practitioners and anyone committed to equitable social and economic development.

Endnotes

^1^Note that capture-recapture was not performed in districts with fewer than 25 children across both counts due to concerns about the precision of the match rate in such settings.

## Abbreviations

DHS: Demographic and Health Survey
MICS: Multiple Indicator Cluster Survey
MoSVY: Ministry of Social Affairs, Veterans and Youth Rehabilitation
